# Targeting Myeloid-Derived Suppressor Cell, a Promising Strategy to Overcome Resistance to Immune Checkpoint Inhibitors

**DOI:** 10.3389/fimmu.2020.00783

**Published:** 2020-05-15

**Authors:** Aohan Hou, Kaiyu Hou, Qiubo Huang, Yujie Lei, Wanling Chen

**Affiliations:** ^1^Faculty of Clinical Medicine, Southwest Medical University, Luzhou, China; ^2^Department of Bone and Trauma, The Second People’s Hospital of Yunnan Province, Kunming, China; ^3^Department of Thoracic Surgery, The Third Affiliated Hospital of Kunming Medical University and Yunnan Cancer Center, Kunming, China

**Keywords:** cytotoxic T-lymphocyte-associated protein 4 (CTLA-4), program death-1 (PD-1), program death-1 ligand 1 (PD-L1), myeloid derived suppressive cells (MDSCs), immune checkpoint inhibitor (ICI)

## Abstract

Immune checkpoint inhibitors (ICIs) are starting to transform the treatment for patients with advanced cancer. The extensive application of these antibodies for various cancer obtains exciting anti-tumor immune response by activating T cells. Although the encouraging clinical benefit in patients receiving these immunostimulatory agents are observed, numbers of patients still derive limited response or even none for reasons unknown, sometimes at the cost of adverse reactions. Myeloid-derived suppressor cells (MDSCs) is a heterogeneous immature population of myeloid cells partly influencing the efficacy of immunotherapies. These cells not only directly suppress T cell but mediate a potently immunosuppressive network within tumor microenvironment to attenuate the anti-tumor response. The crosstalk between MDSCs and immune cells/non-immune cells generates several positive feedbacks to negatively modulate the tumor microenvironment. As such, the recruitment of immunosuppressive cells, upregulation of immune checkpoints, angiogenesis and hypoxia are induced and contributing to the acquired resistance to ICIs. Targeting MDSCs could be a potential therapy to overcome the limitation. In this review, we focus on the role of MDSCs in resistance to ICIs and summarize the therapeutic strategies targeting them to enhance ICIs efficiency in cancer patients.

## Introduction

In the last decades, cancer therapy has been transformed by Immunotherapies whose element is the anti-tumor response mediated by cytotoxic T lymphocyte (CTL). Based on the accumulating data that the tolerant nature of tumor is associated with cancer progression, various methods are proposed aiming to shake the immunosuppressive microenvironment, such as cancer vaccines, adoptively transferred antigen-specific T lymphocytes and Immune checkpoints inhibitors (ICIs). Until today, ICIs are most widely used immunotherapeutic strategies which bind to immune checkpoint molecules inducing the (re)activation of endogenous tumor-specific T-cell immune response. These therapeutic strategies provide an inspiration for the treatment to wide cancer types, such as metastatic melanoma, lung cancer, head and neck cancer, breast cancer, colorectal cancer, hepatocellular carcinoma (HCC), and renal cell carcinoma (RCC) (1–3). Although the encouraging clinical benefits in patients receiving these immunostimulatory agents are observed, therapeutic resistance occurring in numbers of patients limits the application of ICIs leading to ultimately progression (1, 2). Therefore, there is an urgent clinical need to explore the resistance mechanisms of these immunotherapies.

MDSCs is a heterogeneous immature population of myeloid cells halted at multiple stages of differentiation performing the ability to suppress innate and adaptive immune responses (4). Abnormal MDSCs accumulation in patients with advanced cancer is strongly associated with the resistance to immune modification agents (5–7). Furthermore, recent studies suggest the leading role of MDSCs in immunosuppressive tumor microenvironment (TME) which could be a main cause of therapeutic resistance to ICIs (7–10) as the negative correlation between MDSCs and ICIs efficacy (11–17). In cancer patients, the accumulation of MDSCs is observed in peripheral blood, draining lymphoid tissues and tumor sites (18), where they suppress the activation and cytotoxicity of T cell and generate the immunosuppressive networks. They also have been shown contributing to promote the angiogenesis and metastases (19). Thus, targeting MDSCs could be a promising strategy to lead TME reprogramming in combination with ICIs.

In this review, we discuss the phenotypic and functional properties of MDSC, especially the immunosuppressive network they derived. We address the role of MDSCs in resistance to ICIs and summarize the therapeutic strategies targeting them to overcome the limitation of ICIs.

## The Main Phenotype of MDSCs

Mouse MDSCs are classified according to the presence of Gr-1 and CD11b on their membranes. These cells can further be subdivided by Gr-1 into two major groups, cells termed granulocytic or polymorphonuclear MDSCs (PMN-MDSCs), which is phenotypically and morphologically similar to neutrophils, can be defined as CD11b^+^Ly6G^+^Ly6C^*low*^ or CD11b^+^Gr-1^*high*^; cells termed monocytic MDSCs (M-MDSCs) which is phenotypically and morphologically similar to monocytes, can be defined as CD11b^+^Ly6G^–^Ly6C^+^ or CD11b^+^Gr-1^*low*^ (20). These cells are well-defined and consist of myeloid progenitor cells, immature myeloid cells, immature granulocytes, monocytic macrophages, as well as DCs (5).

Compared with murine, human MDSCs are inadequately characterized by no expression of Gr-1 on human leukocytes. The initial notion that MDSCs are solely consisted of immature myeloid cells is being changed due to MDSCs described in recent reports sharing similarities on morphology and phenotype with cells contained more differentiated features (21–23). The overlapping on phenotype and morphology between human M-MDSCs and PMN-MDSCs confuse researcher in depicting their role in human disease. A study implemented by an international consortium including 23 laboratories identified 10 putative subsets of MDSCs in peripheral blood mononuclear cells (PBMC) obtained from healthy donors in pretest based on the marker combination consisted of core markers commonly used by all laboratories (deduce from two webinars), a dead-cell marker, lineage cocktail and CD124. Due to the main variable that the gating strategy, high interlaboratory variance observed in study for all MDSC subsets, especially the granulocytic subsets. As such, further efforts should be made in future studies for defining unique identification of different populations of MDSC through cell-surface markers and gating strategies (24). Recently, a recommendation proposed specific gating strategies and clear procedure for MDSCs identification. The Criteria for the phenotypic characterization of human MDSCs by flow cytometry are now defined as the common myeloid markers expressed (CD14^+^, CD11b^+^, and CD33^+^), HLA-DR^–/*low*^ and low expression of lineage-specific Ags (Lin), such as CD3, CD14, CD15, CD19 and CD56. Three subsets divided from MDSCs have been reported as human M-MDSCs (Lin^–^HLA-DR^*low/*–^CD11b^+^CD33^+^CD14^+^), human granulocytic or PMN-MDSCs (CD11b^+^CD14^–^CD15^+^ or CD11b^+^CD14^–^CD66b^+^) and Lin^–^HLA^–^DR^–^CD33^+^ cells consisted of a mixture of immature progenitors. Since all MDSCs subsets are immature, the third subset has been named as early stage MDSC (e-MDSC) whose existence has yet to be identified in mouse (4). At this point, it appears that each of these cell populations are essential for any characterization of MDSC (25, 26).

The separation of neutrophils from PMN-MDSC now are insufficient because the finite methods and the similarities on phenotype and morphology shared by these cells. Even by Standard Ficoll-gradient centrifugation (at 1.077 g L^–1^ d), the contamination exists between PMN-MDSC and neutrophils. It seems that the functional, biochemical and genomic characterization of PMN-MDSCs described in many studies is conducted for the entire population of cells, not for PMN-MDSCs only. Thus, the precise nature of PMN-MDSC remains vague. Recently, a study identified lectin-type oxidized LDL receptor 1 which is associated with ER stress and lipid metabolism as a marker of PMN-MDSC in humans, but it seems to need more verification by future study (27).

Mononuclear cells have been observed in tumor site performing as a variety of differentiating phases from monocytes or M-MDSCs toward tumor-associated macrophages (TAMs). Phenotypically, M-MDSCs are distinguished from TAM by decreased relative expression of F4/80, but higher expression of S100A9. Compared with M-MDSCs, the elevation of the macrophage terminal differentiation marker, IRF8, and M-CSF receptor, CD115 are available to separate TAM from M-MDSCs (4, 28). A unique subset of M-MDSCs was found contributing to the pool of PMN-MDSCs with identity as monocyte-like precursors of granulocytes. These cells which had limited suppressive activity but potent ability to differentiate to granulocytes obtained the phenotype as CD11b^+^Ly6C^*hi*^Ly6G^–^CD117^+^ monocytic cells with a low Rb1 expression. The selective depletion of monocytic cells including monocyte-like precursors of granulocytes, decreased the population of PMN-MDSCs in tumor-bearing mice more than 50%, whereas no effect on the number of granulocytes in naive mice. In cancer patients, it seemed that CXCR1^+^CD15^–^CD14^+^HLA-DR^–/*low*^monocytic cells are enriched for the population of monocyte-like precursors of granulocytes (29).

It is important to emphasize that the potent immune-suppressive activity of MDSCs is the most reliable marker and main reason to define these cells with the similar morphology and phenotype to neutrophils and monocytes as PMN-MDSC and M-MDSC.

## MDSCs as a Predictive Marker in ICIs for Cancer Treatment

As an important prognostic marker, MDSCs have been widely used in ICI treatment. Patients responded to ipilimumab have been observed significantly lower percentage of M-MDSC in their peripheral blood (11), which is consistent with the study that a high M-MDSC frequency is associated with decreased expansion and activation of tumor-specific T cells (12). Especially in malignant melanoma patients, a lower frequency of circulating MDSC is apparently common trait of clinical responders to ipilimumab treatment (13–15). Furthermore, strong positive correlation between MDSC percentage and neutrophil/lymphocyte rate (NLR), a prognostic marker in both ipilimumab and Nivolumab therapy, have been investigated in patients with breast cancer (30, 31). The research for three cancer types (melanoma, non-small-cell lung cancer, and genitourinary cancer) illustrated that high NLR resulting in a worse overall survival and progression-free survival across a range of ICIs (ipilimumab, nivolumab, pembrolizumab, and nivolumab) (31). Additionally, MDSCs level also is used to predict the clinical outcome of the patients who had failed to ipilimumab and treated with nivolumab. The ipilimumab treatment have no effect on MDSCs function after patients received 12 months ipilimumab accompanying with higher proportion of MDSCs in non-responder, similar to Meyer’s study (11, 16). In prostate cancer treated by the combination of cancer vaccine and ipilimumab, patients who contained high level PBMC activation during treatment and low frequency of M-MDSCs pre-treatment get better clinical benefit, and higher frequency of MDSC in circulation is correlated with reduced overall survival. Meanwhile, despite no correlation with response or survival, the significantly increased frequencies of MDSCs in post-treatment patients were observed (17).

In summary, the MDSCs level is a promising prognostic marker in ICIs therapy. High frequency of circulating MDSCs is associated with low or no response of the patients with ICIs treatment, suggest that the MDSCs may be a key point in resistance occurring in ICIs therapy. Which is need to emphasized is the MDSCs, neither the frequency nor function, are uninfluenced via ipilimumab, unlike the pembrolizumab or nivolumab. A study for biopsies obtained from patients received pembrolizumab, showed that the increased frequency of MDSCs and Tregs in both responders or non-responders and higher percentage of T cells in responders (32). Another study for patients with NSCLC treated by nivolumab have illustrated that the time-depended NLR increasing, which is associated to MDSCs (33). The result of these result indicated that the supplementary strategies targeting these cells during ICIs treatment is necessary.

## MDSCs-Induced Acquired Resistance to ICIs

**FIGURE 1:**
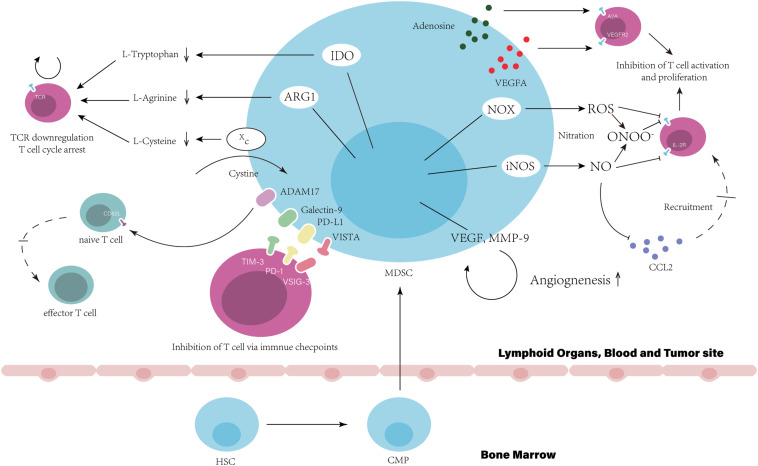
Myeloid-derived suppressor cells suppress T cell function and directly result in the resistance to immune checkpoint inhibitors. In bone marrow, hematopoietic stem cells (HSCs) give rise to common myeloid precursors (CMPs), which then differentiate into MDSCs during tumor progression. The accumulation of MDSCs in tumor site, blood and lymphoid organs, such as the spleen, can be observed when cancer patients are resistance to ICIs. Immune suppression by MDSC is mainly antigen specific, contact dependent, and utilizes several major pathways: (1) Production of reactive nitrogen and oxygen species, such as nitric oxide (NO), reactive oxygen species (ROS), and peroxynitrite (PNT). (2) Elimination of key nutrition factors for T cells from the microenvironment (L-arginine, Ltryptophan, and L-cysteine). (3) Disruption of homing and trafficking of T cells (through the expression of ADAM17, the nitration of CCL2). (4) Production of immunosuppressive eytokines (TGF-3 and IL-10). (5) Upregulation of immune checkpoint, such as PD-Ll, galectin-9, and VISTA. (6) Release of immune regulatory molecules, such as adenosine and VEGFA.

**FIGURE 2:**
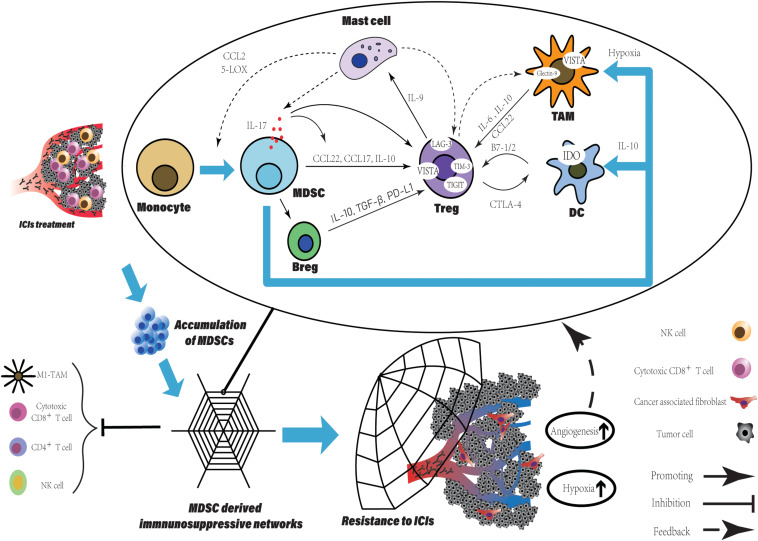
The MDSCs accumulation derived a potent immune suppressive network within TME leading to resistance to ICIs. The accumulation of MDSCs is observed in various tumor type after ICIs treatment and can be a promising predictive marker. Besides the direct suppression of T cell function, MDSCs participate in crosstalk between not only immune cells, but immune cells and non-immune cells. Within TME, such crosstalk generates positive feedback loops to reinforce the suppressive immune network, which is an amplifier to extend the intrinsic immune regulation function of MDSCs and to augment their pro-tumorigenic effects. More immune suppressive cells are recruited and induced with various immune checkpoints expression. Finally, TME has been reprogrammed into limited anti-tumor immune response induced by ICIs, companied with increased level of tumor angiogenesis and hypoxia which enhanced the network.

The suppressive activity of MDSCs is mediated by multiple mechanisms. The suppression derived from MDSCs disrupt T cell normal function via direct cell-cell contact or indirect effect on remodeling of the microenvironment. Here, we summarize the functional characteristics of MDSCs and divide them into three ways contributing to resistance to ICIs: (1) Target T-cell directly that lead to T-cell dysfunction (Figure 1). (2) Inherent ability to promote tumor angiogenesis. (3) Structure the TME through the cellular and molecular immunosuppressive network mediated by MDSCs (Figure 2).

### Target T-Cell Directly That Lead to T-Cell Dysfunction

As the main target of ICIs, T-cell function is critical for patients response to ICIs and impaired by MDSCs directly via cell-cell contact or unique expression pattern, which is associated with poor clinical outcome of patients with ICIs treatment (34). High expression level of arginase I (ARG1) in MDSCs have been shown in some studies that starves L-arginine then leading to T-cell cycle arrest and T cell dysfunction via the downregulation of T cell receptor (TCR) ζ-chain expression (35, 36). By expression of Xc transporter, cysteine is forfeited by MDSCs resulting in the blockade of T-cell activation through limited availability of cysteine (37). The reduction of TCR function also can be generated by the hyperproduction of reactive oxygen species (ROS) and peroxynitrite form MDSCs, which results in the nitration of TCR/CD8 during direct cell contact between MDSCs and CD8^+^T-cell, then impairs the ability of CD8^+^T cells to bind the specific peptide MHC and respond to the specific peptide (38). Similarly, the nitration by MDSCs-derived peroxynitrite has been reported that influenced chemokine CCL2, which subsequently affected T cell migration (39). NO produced by MDSCs impairs T cell of signaling pathway activated by the IL-2R, inhibits the mitogenic and peptide-specific responses (40). MDSCs-expressed cell surface ADAM17 downregulates L-selectin on naïve CD62L^+^T cells decreasing their migration into effector sites, which results in attenuated expansion and activation of effector T cells (Teffs) (41). Proangiogenic factors released by MDSCs also contribute to the inhibition of T cell. Ziogas et al. demonstrated that the vascular endothelial growth factor A (VEGFA) suppresses activation of T cells via VEGF receptor 2 (VEGFR2) (42). MDSC can also produce immunosuppressive cytokines, such as IL-10 and TGF-β (7). In addition, the abnormally high levels of CD39/CD73 expressed on MDSCs lead to the release of adenosine, which subsequently inhibits T cell proliferation mainly via A2A receptor activation resulting in the reduction of the antitumor immune responses (43). Indoleamine 2, 3-dioxygenase (IDO) shares the similar mechanism with ARG1 and limits the effect of ICIs (44). The SATA3-depended expression of IDO in MDSCs have been demonstrated that mediated the inhibition on T cell proliferation and Th1 polarization, and promotion of T cell apoptosis and secretion of immunosuppressive cytokines in breast cancer (45). IDO expression depletes tryptophan and produces kynurenine-based metabolites in the microenvironment suppressing T cell proliferation and inducing T cell apoptosis via the GCN2 pathway (46). Also, IDO is responsible to the impairment of immune surveillance and derive the immune tolerance by suppressing TCR-mediated activation of T cells (47).

After the initial administration using ICI treatment, the secondary upregulation of multiple immune checkpoints expressed on MDSCs is an important mechanism impairing efficiency of ICIs. In tumor site, MDSCs show an increased level of PD-L1 induced by hypoxia and the exposure of splenic MDSC to hypoxia resulted in similar upregulation of PD-L1 (48). The highly increased number of TIM-3 expressing CD4^+^ and CD8^+^ T cells have been observed in samples from lung cancer patients and murine model with acquired resistance to anti-PD-1 treatment (49). Cytotoxic CD8^+^T cell in non-small-cell lung cancer (NSCLC) patients induced anti-tumor response initially and highly expressed TIM-3 after treatment of anti-PD-1 mAb. This response to anti-PD-1 mAb of patients were reversed through the binding of TIM-3 expressed on T cell and galectin-9 (TIM-3 Ligand) on MDSCs (34). Recently, V-domain Ig suppressor of T-cell activation (VISTA) is proposed to be an immune checkpoint protein highly expressed on MDSCs mediating suppression of T cell response (50). Hypoxia contributed to high expression of VISTA on MDSCs (51). Gao et al. found that elevated level of VISTA in patients with prostate cancer after ipilimumab therapy, (52) which binds to its ligand, V-Set and Immunoglobulin domain containing 3 (VSIG-3) suppressing Teffs proliferation and activation (53). The co-blockade of PD-L1 and VISTA in mouse tumor models maximizes tumor-clearing therapeutic efficacy, suggests that VISTA is a potential mechanism that improves the resistance to ICIs (54).

### Inherent Ability to Promote Tumor Angiogenesis

Myeloid cells have been studied to be drivers of angiogenesis. Although unusual metabolic pattern is displayed in MDSCs with critical role in cancer immune modification, the heritage of myeloid cells that promoting angiogenesis are contained by those cells (55). The neutralization of BV8-specific antibody decrease the number of MDSCs in tumor-bearing mice and has dramatically reduced angiogenesis, suggest that MDSCs in this process (56). A very well studied molecule in tumor angiogenesis, VEGFA can be released by MDSCs (57). This secretion is ligand for angiogenic receptor, VEGFR2, a tyrosine kinase receptor expressed on nearby endothelial cells (EC). The activation of VEGFR2 promotes ECs proliferation through the PLCγ-PKC-MAPK signal pathway (58). The binding to cell adhesion molecule (CAM)-family proteins on ECs extravasate T cells to tumors. Myeloid cells-derived VEGFA and FGF2 influenced ECs of tumor-associated blood vessels downregulating their expression and separating intercellular adhesion molecule 1 (ICAM1) and vascular cell adhesion molecule 1 (VCAM1), which may impose restrictions on T cell adhesion and extravasation, finally impairing T cell homing to tumors (59–61). In addition, the VEGFR2 on myeloid cells results in the malignant progression of gliomas in mice. The upregulation of VEGFR2 mediated by ID2 promotes the direct differentiation of pro-angiogenic myeloid cells and is critical for their angiogenic function. Knockdown of ID2 shows that fewer MDSCs are recruited to tumors (62). This finding suggests that VEGFA-VEGFR2 signaling in MDSCs may be a positive feedback promoting their differentiation and possibly increasing the secretion of pro-angiogenic factors within TME. VEGFA suppress the differentiation of CD34^+^ hematopoietic progenitors into mature DCs via VEGFA-VEGFR1 signaling pathway resulting in antigen presentation defect. VEGFA also induces PD-L1 expression on DCs (63). In HCC mouse model, β-catenin activation promotes the immune escape mediated by a defect in DC recruitment and shows the resistance to anti–PD-1 therapy (64). Hammerich et al. demonstrated that recruiting and activating intratumoral, cross-priming DCs regress and potentiate PD-1 blockade (65). In contrast to VEGFA directly suppress T cell proliferation that we mention above, these findings further indicate that lack of DCs and impaired antigen presentation may be a potential mechanism of resistance to ICIs, which can be caused by VEGFA-VEGFR1 signaling pathway activation.

In various tumor model, the secretion of matrix metallopeptidase 9 (MMP-9) in MDSCs has been reported (66–68). MMP-9 has been shown to function as an angiogenic switch and release VEGF from the extracellular matrix. MDSCs utilize this way to regulate VEGF bioavailability in tumors, for example, inducing significantly more VEGF releasing in tumor and ECs migration (66, 69). Interestingly, some ECs marker can be expressed on MDSCs, such as vascular endothelial cadherin (VE-cadherin), the necessary adhesion molecule for clustering ECs in the vasculature (70). During angiogenesis, a research observed myeloid cells recruitment near the EC wall of blood vessels. The congregation of MDSCs around existing vasculature presumably indicating that cell-surface VE-cadherin expressing may be utilized, together with secretion of pro-angiogenic factors, making them very efficient and potent propeller for angiogenesis (71).

There are two essential mechanisms for angiogenesis induced by MDSCs that seem to be responsible for the resistance to ICIs. The first one is the immunosuppressive properties of these pro-angiogenesis secretions we have discussed. Another one is the distinctive vasculature with structural and functional defect generating under overexpressed pro-angiogenic factors. The abnormal tumor vasculature shows very slow, inefficient blood flow which makes drug delivery more difficult. This barrier has been observed in chemotherapy and is reversed by normalizing the tumor vasculature via deletion of myeloid-derived VEGFA and it also should be considered in ICIs (72). Addtionally, the pathological blood flow passed through these vessels is unable to support cell metabolism. Hypoxia within TME has been demonstrated widely that facilitates the recruitment of immunosuppressive myeloid cells, partly, remodels the TME toward immunosuppressive, which subsequently indicates that the angiogenesis derived by MDSCs is a positive feedback to magnify their impact on immune microenvironment (55). By hyperoxygenation of TME, a research showed increased CTLs activity and improved clinical responses to ICIs (71). It suggests that the combination of immune therapies and strategies which normalize tumor vasculature attenuates the hypoxic TME, thus promoting the function of T cells. More data from pre-clinical or clinical study will provide the evidence about the efficiency of these strategies and answer the question that if they could break the positive feedback loop.

### Structure the TME Through the Cellular and Molecular Immunosuppressive Network Mediated by MDSCs

Various cells within TME, such as immune, stromal, endothelial and cancer cells, communicate with each other to create an immunosuppressive network. MDSCs participate in the crosstalk between not only immune cells, but immune cells and non-immune cells generating positive feedback loops, finally leading to acquire resistance to ICIs. As an “amplifier,” this mechanism extends the intrinsic immune regulation function of MDSCs and to augment their pro-tumorigenic effects.

Firstly, we describe the link between regulatory T cells (Tregs) and MDSCs. As we know, the accumulation of MDSCs is associated with the immune tolerance to tumors. Tregs expansion is directly induced by expression of CD40 on MDSCs (73). IL-17 released by MDSCs not only promotes the Tregs infiltration via stimulating MDSCs to produce CCL17 and CCL22, but also enhances the suppressive function of Tregs by increasing the expression of CD39 and CD73 on Tregs (74). Using human M-MDSCs induced by prostaglandin-E2 showed more potent ability of immune suppression and promoting expansion of IL-10-producing cells, especially Tregs (75). Tregs recruit mast cells into tumor site via secretion of IL-9 by themselves under stimulation of mast cell, meanwhile, IL-9 maintains the immunosuppressive ability of Tregs and promote tumor progression (76). In turn, mast cells are responsible for the trafficking monocytes into the TME, the induction of monocytes differentiation into MDSCs and the stimulation of suppressive function of MDSCs through CCL2 secretion and 5-lipooxygenase (5-LOX) upregulation (77). Finally, the MDSCs-derived IL-17 triggered by mast cells to complete the feedback loop including MDSCs, Tregs and mast cells (74).

Opposite roles like T cells of B cells, either enhancing or suppressing anti-tumor immunity have been reported (78). Suppressive B cells called Bregs are transformed from normal B cell. After co-culture with isolated MDSCs from breast tumor, B cells perform an unique phenotype with expression of PD-L1 and production of IL-10 (79). Furthermore, the highly secretion of IL-10 and TGF-β from Bregs promotes Tregs activity and expansion within the TME (80–82). Bregs-derived PD-L1, which binds to PD-1^+^Tregs promotes Tregs proliferation suppressing the anti-tumor response and leading to cancer progression (83).

It needs to be emphasized that the further differentiation of M-MDSCs is seemed to be abnormal to the general differentiation of myeloid cells. In tumor site, M-MDSCs rapidly differentiate into TAMs compared to those in spleen. By intravenous injection, M-MDSCs were transferred directly into either tumor site or spleen in the same EL-4 tumor-bearing mice. Splenic M-MDSCs differentiated slowly to macrophages, whereas in tumor these cells rapidly differentiated to TAMs (84). This result is consistent with recent studies in various tumors, MDSCs are a predominant population of myeloid in spleen contrast to in tumor, TAMs is more frequent (85). Additionally, hypoxia within TME facilitates the differentiation of M-MDSCs and diverts it toward immunosuppressive TAMs, closer to M2-macrophage (84, 85). A crosstalk between MDSCs and macrophages via cell contact reduces the IL-12 production in macrophages, whereas increases IL-10 production in MDSCs, indicates that MDSCs alter the phenotype of macrophages toward M2 polarization and induce Tregs expansion (86). TAMs communicate with Tregs and generate a positive feedback loop. IL-6 released by TAMs induces the production of IL-10 by tumor cell via SATA3 signaling, which further enhances the IL-10 level within TME facilitating Tregs activation, survival, and accumulation (87). The combination of IL-6 produced by TAMs and elevated level of IL-10 is contributing to Tregs activation. Furthermore, TAMs secrete CCL22 to traffic CCR4^+^Tregs into tumor site (88, 89). In turn, Tregs induce monocytes differentiating into macrophages with a TAM-like phenotype via producing IL-10 or cell contact (90). M-MDSCs also inhibited NK-cell activity through the production of TGF-β (91). As another direction of M-MDSCs differentiation, DCs, is inhibited at the stage of differentiation and gain an immune regulatory phenotype. Not only hypoxia and VEGFA can be the causes of blocking the differentiation into DCs, but there is evidence that IL-10 derived from PMN-MDSCs involve in the suppression (63, 92, 93). High level of IL-10 within the TME drives DCs toward regulatory function that low immunostimulatory molecules expression and high suppressive cytokines production (94).

Increased level of immune cells is also responsible for promoting tumor angiogenesis. Various cells (TAMs, Tregs, MDSCs, cancer-associated fibroblasts, Neutrophils, and mast cells) participate in process of tumor angiogenesis by inducing ECs proliferation, migration and survival, as well as extracellular matrix (ECM) remodeling, breaking the balance of non-angiogenic TME toward angiogenic TME via production of pro-angiogenesis factor (55). As we have discussed above, tumor angiogenesis augments the hypoxia within TME, which subsequently supports immunosuppressive cells recruitment and differentiation. Hypoxia stabilizes hypoxia-inducible factor (HIF)-1/2α dimerizing with HIF-1β to initiate gene transcription through binding to target genes (95). Chiu et al. demonstrated that the hypoxic regions in tissues from human HCC were infiltrated by MDSCs preferentially depending on HIFs expression. The HIFs activate the transcription of CCL26 in cancer cells to recruit CX_3_CR1^+^MDSCs and prevent the differentiation of M-MDSCs into non-immune suppressive CD11c^+^DCs via upregulation of extracellular 5′-AMP (92, 96). Similarly, Tregs and TAMs are recruited to and retain inside hypoxic niche by expression of CCL28 and Sema3A/Nrp1 signaling respectively (97, 98). Furthermore, the hypoxic TME enhanced the production of tumor-derived extracellular vesicles (EVs), which is an important pattern for tumor to evade immune surveillance. By secretion of EVs, ECM remodeling and tumor stromal cells are induced (99, 100). For stromal cells, CAFs is one of the dominators within TME in many types of solid tumor converted from normal resident fibroblasts. The Treg:CD8+ T cell ratio is increased by the interaction between CAFs and tumor cells via the CAFs-derived IL-6 (101). Using co-culture systems, the crosstalk between breast cancer cell and fibroblasts upregulated cytokines, such as IL-6 IL-8 and TGF-β and chemokines, such as CXCL1 and CXCL3, which are the molecules involving in the migration of cells and the induction epithelial-mesenchymal transition (EMT) (102). As one secretion of Immunosuppressive intratumoral Tregs (103), TGF-β also is essential for Tregs about inducing FoxP3 expression, cell differentiation and maintenance, and their suppressive activity (104). Furthermore, the effect of TGF-β on normal resident fibroblasts leads their differentiation toward CAFs (105).

MDSCs-induced immunosuppressive network within TME is a barrier for the anti-tumor response extending in cancer patients and limit the application of ICIs. The direct result of the network is elevated level of immunosuppressive cells recruitment and accumulation, such as TAMs, Tregs, and MDSCs themselves (73, 77, 90). Also, some immune cells gain a inverse phenotype showing the immunosuppressive function, such as DCs, T cells, TAMs and B cells (79, 86, 94, 106). A vast array of molecules continuously produced by these cells inhibits anti-tumor immunity and promotes tumor progression and metastasis (7, 107), for example, TGF-β and IL-10, which is involved in suppression of the MHC II molecules expression, APCs maturation and inhibition of Teffs on differentiation, proliferation, and function (108, 109). The immune regulation function of these cells even exists in dead cells. Apoptotic Tregs elevates the level of extracellular adenosine, which also secreted by MDSCs and TAMs, subsequently influencing Teffs to suppress their proliferation and function and inducing the acquired resistance to anti-PD-L1 treatment (43, 110). Similar to MDSCs, the overexpression of immune checkpoint on these cells is complementary mechanism for the acquired resistance to ICIs. Tregs within the TME express the increased levels of other immune checkpoints besides PD-1 and CTLA-4, namely TIM-3, LAG-3, VISTA and T-cell Ig and ITIM domain (TIGIT) (111). Upregulated level of VISTA on tumor-infiltrating lymphocyte and M2-macrophages have been illustrated in patients with prostate cancer who received the treatment of anti-CTLA-4 mAb (52). DCs and MDSCs highly expressed VISTA and regulated their tumor effector function via controlling the activation of MAPKs and NF-κB (112). The frequency of galectin-9^+^TAMs predicted poor overall survival and recurrence-free survival, is correlated with tumor stage and grade in bladder cancer patients. Galectin-9^+^TAMs reduced the anti-tumor response of immune cells and promoted tumor growth via T cell exhaustion (113). The co-blockade of CTLA-4 and LAG-3 or PD-1 and LAG-3 showed better therapeutic efficacy through increased CD8^+^T cells with/without the reduction of Tregs in the tumor, compared to single CTLA-4 or PD-1 targeting (114). Zhang et al. reported the blockade of TIGIT sensitized the mouse tumors to anti-PD-1 antibodies (115). The more beneficial outcomes that high survival have been observed in tumor-bearing mice received co-blockade of PD-1 and TIGIT treatment, compared to those treated with single anti-TIGIT mAbs (116). Cells involved in immune suppressive network is a force to be reckoned with during the tumor angiogenesis, due to their pro-angiogenesis function. The problems we discussed above, such as the overexpression of pro-angiogenesis factors, “messy” vasculature and hypoxia still impact on ICIs resistance, even worse because the amplification through complicated crosstalks.

In summary, these data suggest more potent immunosuppressive TME is induced by not only MDSCs, but other cells influenced by MDSCs. Through the network, such mechanisms like the upregulation of ICs, tumor angiogenesis, T cell dysfunction are seemed to become more powerful to produce the acquired resistance to ICIs due to increased numbers and type of immune and non-immune cells. It suggests that targeting MDSCs, the important net point that supports the network is a feasible potential strategy to overcome ICIs resistance.

## Different Role of MDSCs, Solid Cancer Versus Hematological Cancer

The widely defined fact that solid cancer harbor the differently pathological setting comparing to hematological cancers is crucial for immunotherapy. The clinical respond rates (RR) of patients with solid tumor are generally lower than hematological cancer (about 10–30% vs. 36–87%), reviewed by Hamanishi et al. (117). In a clinical trail which enrolled 23 patients with relapsed or refractory Hodgkin’s Lymphoma, 87% objective respond rate was observed containing 17% complete response (118). To answer how the hematological tumor get such dramatic anti-tumor response that solid tumor can’t, it may derived from several aspects as follow. First of all, the co-circulation of antibodies and cancer cells in hematological is beneficial for the blockades to exert effect, whereas the hostile TME within solid tumors as we described above is too powerful to it. That special character of these type tumors helps drugs bypassing vascular barrier and stromal hinder. For example, Sun et al. used the nanoparticles comprising of PD-L1 recognizable peptide DPPA-1 and the sequence of CGKRK (a tumor vasculature affinity peptide) to form a unique CD peptide. By delivery through another nanoparticles, paclitaxel-loaded, the synthesis CD peptide showed improved cytotoxicity and inhibition for angiogenesis. The more important result was the single PD-L1 affinity peptide decorated group was less efficient than CGKRK decorated alone group, which the latter is nearly equal to co-decorated group, suggesting the normalizing of vasculature probably more elementary in anti-cancer effect (119). Moreover, study have illustrated that ECM is responsible for inertia of solid tumors to ICIs. In metastatic melanoma, the positivity of α-smooth muscle actin (α-SMA), the crucial composition of the ECM, is negatively correlated with clinical outcome in patients after anti-PD-1 treatment. Additional, another crucial ECM composition, fibroblast-activated protein (FAP) can be a negative prognostic biomarker before the immunotherapy in this case (120).

We easily find that these mechanisms making solid cancers insensitive to immunotherapy can be enabled by MDSCs. This barrier is common in much therapies, for instance, chimeric antigen receptor modified T-cell (CAR-T) therapy (121). What’s more, MDSCs also play a non-negligible role in various hematological cancers, such as lymphoma, leukemia and multiple myeloma. Lv and Wang et al. reviewed the MDSCs in hematological cancers of frequency, characters and mechanisms. Besides the general ways, such as upregulation of arginase 1, ROS, iNOS, secretion of IL-10, TNF-β and enhanced expression of PD-1 and VISTA, the mechanisms of MDSCs we have mentioned in solid cancers to suppressed the T cell function, PMN-MDSCs in multiple myeloma express higher levels of PROK2 digesting the bone matrix. Higher MDSCs level is observed in hematological cancer patients and those of advanced stage or relapsed, in contrast to health donor (122). The study of diffuse large B-cell lymphoma (DLBCL) revealed that MDSCs-induced T cell suppression is partial attributed to PD-L1 expression and is restored by monocyte depletion. The number of M-MDSC is associated to negative parameters, such as International Prognostic Index, event-free survival, and number of circulating Tregs. The result established by Wilcox et al. in B cell-derived non-Hodgkin lymphoma further proved it (123). Similar situation occurring in high Sokal risk level patients with chronic myeloid leukemia, where the MDSCs level is high accompanying with upregulated expression of PD-1/PD-L1 on T cells (124). Wang et al. have investigated that declined T cell disfunction via knockdown of VISTA, which is highly expressed on MDSCs in acute myeloid leukemia with higher cell number (50). One review have illustrated the effect of MDSCs on immunotherapies, not only ICIs, but cancer vaccine and CAR-T. As for ICIs, MDSCs in multiple myeloma harbor higher level of PD-L1 expression than antigen-presenting cells, are inhibited by PD-1/PD-L1 blockade directly (125).

Although the studies for the changing of MDSCs after the ICIs treatment are remain rare, the studies we listing above may suggesting the combination of ICIs and anti-MDSCs strategies is a feasible method. But, it is need to be emphasized that the combination strategies should be cautious, because hematopoietic stem cell transplantation is a routine and efficient therapy in hematological cancers treatment. The balance among the graft versus leukemia, graft versus host disease (GVHD) and MDSCs effect in hematological tumors is fragile and exquisite. MDSCs deemed as a protector in the balance, differentiating into MHC class II ^+^CD80/CD86^+^CD40^–^ cells to promote transplantation tolerance by IL-10 downregulation, Tregs reduction. Meanwhile the graft versus leukemia effect is keeping (126). In clinical, higher level of MDSCs has been observed in patients suffering GVHD, in contrast to patients received graft without GVHD. G-CSF is responsible for the MDSCs expansion, which the latter is negatively associated with the risk of GVHD in allo-HSCT. Systemic G-CSF treatment showed the effect on expansion of both M-MDSCs and e-MDSCs, which can predict the acute and chronic GVHD, without distinct impact on survival and relapse (127, 128). This result may partially explain why the MDSCs-targeting treatment in hematological cancers is lack. However, the role of MDSCs in this specific setting is remain controversial, especially of the reconstituted MDSCs.

## Combination of ICI and MDSCs Targeting

Recently, the combination of ICIs treatment with MDSC targeting has been shown a surprising effect when applied in preclinical cancer model or cancer patients. Strategies that targeting MDSCs impact on expansion, trafficking, and inhibition function, break the chain of immune-modulatory reaction after ICIs administration (Table 1). Interestingly, PBMC isolated from cancer patients seems to be directly stimulated by anti-PD-1 antibodies. In vitro, anti-CD3 antibodies-induced PBMC proliferation are activated via the stimulation of PD-1 blockade, but the inhibition of MDSC in the same experimental settings (129).

**TABLE 1:** Combination therapy of myeloid derived supressor cells (MDSCs) targeting with immune checkpoint inhibitors (ICIs).

No.	Conditions	Interventions	Effect	References
**ICI plus reduction of MDSC frequency**				
1	SCLC mouse model	Low doses gemcitabine with SRA737 +anti-PD-1	Decreased MDSCs population, regressed tumor	(139)
2	RCC cell lines and mouse model	5-FU+anti-PD-L1	Enhanced ratio of CD8^+^ immune cells and CD11b^+^Ly6G^+^Ly6C^*low*^ MDSC, prolonged survival time and Improved survival	(142)
3	BRAF V600E/PTEN-null melanoma mouse model	Phenformin+anti-PD-1	Reduced the proportion of GMDSCs in the spleens of tumor-bearing mice., increased the level of ROS reaching toxic threshold level in G-MDSCs, decreased the expression of arginase 1, S100A8, and S100A9, inhibited tumor growth	(144)
4	Tgfbr1/Pten 2cKO mouse model	Dasatinib+anti-CTLA-4	Decreased MDSCs, inhibited tumor growth and tumor cell proliferation	(145)
5	CCRK-inducible transgenic mice and Hepa1–6 orthotopic HCC models	Genic CCRK depletion+anti-PD-L1	Reduced tumor-infiltrating MDSCs, eradicated large hepatoma	(146)
6	RMS mouse model	Genic CXCR2 depletion+anti-PD-1	Prevention of MDSC trafficking, improved overall survival	(147)
7	KRAS^*G*12*D*^ CRC mouse model	CXCR inhibitor SX-682+anti-PD-1	Reduced MDSCs in the spleen of mice bearing, extended survival time	(149)
8	TH-MYCN murine neuroblastoma model	Selective CSF-1R inhibitor BLZ945+anti-PD-1/L1	Reduced MDSCs in the spleen of mice bearing, reactivated macrophages in spleens, inhibited tumor growth	(151)
9	B16-IDO melanoma mouse model	CSF1R inhibitor PLX647+anti-CTLA-4/PD-1	Depleted suppressive MDSCs, delayed tumor growth	(152)
10	CT26 colon and 4T1 breast cancer mouse models	Anti-CSF1R Abs CS7+anti-CTLA-4	Reduced the number of M-MDSCs, reprogrammed M-MDSCs, delayed tumorgrowth with prolonged survival	(150)
11	PDAC mouse model	CSF1R inhibitor PLX3397/GW2580+anti-CTLA-4/PD-1	Reduced the number of M-MDSCs, blocked tumor progression and even regressed tumor	(153)
**ICIs combined with an alteration of MDSC function**				
1	RCC and NSCLC mouse model	Entinostat+anti-PD-1	Downregulation of ARG1, iNOS and COX-2, inhibits tumor growth	(156)
2	B16F10 melanoma tumor and breast mouse model	Ibrutinib+anti-PD-L1	Reduced frequency of MDSCs, attenuated NO production and IDO expression, inhibited tumor growth	(157)
3	KRAS-mutant CT26 mouse colorectal cancer model	Selumetinib+anti-CTLA-4	Reduced frequency of CD11^+^Ly6G^+^myeloid cells, differentiated MDSCs	(166)
4	Stage III or stage IV melanoma patients	ATRA+Ipilimumab	Reduced the expression of the immunosuppressive genes NOX1, IL10, TGF (3, IDO, and PDL1 and the frequency of circulating MDSCs, increased the expression of the C II TA and the frequency of HLA-DR(+) myeloid cells, prevented tumor progression	(170)
5	Glioblastoma mouse model	Aflibercept+trebananib+anti-PD-1	Reduced tumor-promoting MDSCs, significantly normalized global vessels and extended survival	(171)
6	Melanoma brain metastases model	Axitinib+anti-CTLA-4	Increased number of MDSCs with higher ratio of M-MDSCs and PMN-MDSCs, reduced suppression function of MDSCs, induced antigen-presenting function of M-MDSCs in subcutaneous tumor, reduced tumor growth and increased survival	(172)
7	Head and neck cancers mouse model	IPI-145+anti-PD-L1	Reduced the production of ARG1 and iNOS in PMN-MDSCs, significantly enhanced tumor growth control and survival	(173)
8	CT26 tumor mouse model	QA+anti-PD-1	Reduced the expression of Arg1 and Nos2 transcript levels, slowed tumor growth and increased survival time	(174)
**Clinical trial**				
No.	NCT Number	Tittle	Conditions	Interventions
1	NCT04193293	A Study of Duvelisib in Combination With Pembrolizumab in Head and Neck Cancer	Head and Neck Squamous Cell Carcinoma	duvelisib pembrolizumab
2	NCT04118855	Toripalimab Combined With Axitinib as Neoadjuvant Therapy in Patients With Non-metastatic Locally Advanced Nonmetastatic Clear Cell Renal Cell Carcinoma	Nonmetastatic Locally Advanced Renal Cell Carcinoma	Axitinib Toripalimab
3	NCT03959293	Clinical Trial Evaluating FOLFIRI + Durvalumab vs. FOLFIRI + Durvalumab and Tremelimumab in Second-line Treatment of Patients With Advanced Gastric or Gastro-oesophageal Junction Adenocarcinoma	Gastric Adenocarcinoma Gastric Cancer	FOLFIRI Protocol Tremelimumab Durvalumab
4	NCT03768531	Safety and Tolerability Study of Nivolumab and Cabiralizumab for Resectable Biliary Tract Cancer	Resectable Biliary Tract Cancer	Nivolumab Cabrilizumab
5	NCT03736330	A Study of Anti-PD-1 Combinations of D-CIK Immunotherapy and Axitinib in Advanced Ranal Carcinoma	Renal Cancer Metastatic	D-CIK anti-PD-1 Axitinib
6	NCT03581487	Durvalumab, Tremelimumab, and Selumetinib in Treating Participants With Recurrent or Stage IV Non-small Cell Lung Cancer	Recurrent Lung Non-Small Cell Carcinoma Stage IV Lung Cancer AJCC v8 Stage IVa Lung Cancer AJCC v8 Stage IVb Lung Cancer AJCC v8	Durvalumab Selumetinib Tremelimumab
7	NCT03516279	Pembrolizumab and Dasatinib, Imatinib Mesylate, or Nilotinib in Treating Patients With Chronic Myeloid Leukemia and Persistently Detectable Minimal Residual Disease	Chronic Phase Chronic Myelogenous Leukemia, BCRABL1 Positive Minimal Residual Disease	Dasatinib Imatinib Mesylate Nilotinib Pembrolizumab
8	NCT03332498	Pembrolizumab in Combination With Ibrutinib for Advanced, Refractory Colorectal Cancers	Colon CancerColorectal Cancer Colorectal Carcinoma Colon Disease	Pembrolizumab Ibrutinib
9	NCT03202758	Evaluation of the Safety and the Tolerability of Durvalumab Plus Tremelimumab Combined With FOLFOX in mCRC	Colorectal Cancer Metastatic	Durvalumab Tremelimumab FOLFOX
10	NCT03086174	Tolerability and Pharmacokinetics of Toripalimab in Combination With Axitinib in Patients With KidneyCancer and Melanoma	Kidney Cancer Stage IV Advanced Melanoma	anti-PD-1 Toripalimab
11	NCT02936752	Entinostat and Pembrolizumab in Treating Patients With Myelodysplastic Syndrome After DNMTiTherapy Failure	Myelodysplastic Syndrome	Entinostat Pembrolizumab
12	NCT02750514	An Investigational Immunotherapy Study to Test Combination Treatments in Patients With AdvancedNonSmall Cell Lung Cancer	Advanced Cancer	Nivolumab Dasatinib Relatlimab Ipilimumab
13	NCT02551159	Phase III Open Label Study of MEDI 4736 With/Without Tremelimumab Versus Standard ofCare (SOC) in Recurrent/Metastatic Head and Neck Cancer	Squamous Cell Carcinoma of the Head andNeck	MEDI4736 Tremelimumab 5-FU Cetuximab Cisplatin Carboplatin
14	NCT02526017	Study of Cabiralizumab in Combination With Nivolumab in Patients With Selected AdvancedCancers	Advanced Solid Tumors, Including But Not Limited to Lung Cancer	Nivolumab cabiralizumab
15	NCT02332980	Pembrolizumab Alone or With Idelalisib or Ibrutinib in Treating Patients With Relapsed or RefractoryChronic Lymphocytic Leukemia or Other Low-Grade B-Cell Non-Hodgkin Lymphomas	Recurrent Chronic Lymphocytic LeukemiaMultiple Lymphoma	Ibrutinib Idelalisib Pembrolizumab
16	NCT01928576	Phase II Anti-PD1 Epigenetic Therapy Study in NSCLC.	Non-Small Lung Cancer, Epigenetic Therapy	Nivolumab Entinostat Azacitidine

### ICI and Reduction of MDSC Frequency

Under the pathological conditions, MDSCs are increased in abundance. There is a two-phase model to describe this process, proposed by Condamine and Gabrilovich. The first phase contains the expansion of immature myeloid cells correlated with the blockade of terminal differentiation from hematopoietic stem cells toward granulocytes, macrophages, or DCs, and the second phase involves in activating immature myeloid cells to MDSCs (130, 131). Despite the dominant factors of these two phases overlapping significantly, growth factors mostly derived by tumor, such as GM-CSF, G-CSF prefer to govern the first phase, whereas the proinflammatory cytokines produced by tumor stroma are dominator in the second phase, such as IL-1b, IL-6, and TNF-a (132). As thus, the reduction MDSC frequency need to normalize the procedure of myelopoiesis and block the accumulation of MDSC.

Some chemotherapeutic drugs showed the effect on MDSCs in tumor-bearing hosts. Gemcitabine is a nucleoside analog decreasing the level of splenic MDSC. Thus, research combining gemcitabine with IFN-β showed enhanced antitumor activity of IFN-β (133). In mesothelioma, Gemcitabine synergizes with ICIs showed better efficacy than gemcitabine or ICIs as monotherapy in mice and overcome the resistance to ICI in patients (134). Adding gemcitabine chemotherapy to SRA737 (an oral CHK1 inhibitor) improves the efficacy of PD-L1 blockade for small cell lung cancer. It is associated with the decrease of MDSCs, Tregs, as well as PD-1^+^/TIM-3^+^ exhausted CD8^+^T cells, and the increased M1:M2 macrophages ratio (135). The pyrimidine analog 5-FU has widespread cytotoxicity of cells in vivo, including MDSCs (136). However, 5-FU induces Nlrp3 inflammasome, which promotes the secretion of IL-1b by MDSCs and angiogenesis (136, 137), the RCC xenograft tumor-bearing mice received the combination treatment of 5-FU and anti-PD-L1 Abs has survival time and survival improvement, compared to those who received single treatment of 5-FU or anti-PD-L1 Abs (138). Paclitaxel in ultra-low non-cytotoxic dose reduces both the number and immunosuppressive activity of MDSC, leading to increased survival of melanoma-bearing mice through p38 MAPK and S100A9 signaling (139). An ongoing clinical trial (NCT02425891) of atezolizumab in combination with nab-paclitaxel for patients with previously untreated metastatic triple-negative breast cancer will provide more evidence about the efficacy and safety.

Furthermore, phenformin, an antidiabetic biguanide class drug, inhibited MDSC of significantly decreased proportion of PMN-MDSCs and reduction of ARG1, S100A8 and S100A9 enhancing the efficacy of PD-1 blockade, which is reflected in more infiltration of CD8+ T cell in the BRAF^*V600E/null*^ melanoma mouse model (140). Dasatinib, a SRC family kinase inhibitor, facilitated anti-CTLA-4 immunotherapy in head and neck squamous cell carcinoma by decreasing MDSCs population and increasing CD8^+^T cell:Treg ratio (141). The inhibition of hepatoma-intrinsic cell cycle-related kinase (CCRK) in combination with the anti-PD-L1 antibody was resulted in a significant reduction in the percentages of tumor-infiltrating PMN-MDSCs and M-MDSCs, which were accompanied by markedly increased cytotoxic IFN-γ^+^TNF-α^+^CD8^+^ T cells, suggested that the co-blockage enhanced the efficacy of anti-PD-L1 in HCC via abrogation of MDSC (142).

Although the capability that depleting MDSCs in dose-dependent way, chemotherapeutic drugs is insufficient, which indicates that the MDSCs with lower frequency are still immunosuppressive in tumors. Approaches combining immunotherapy with agents that block MDSCs trafficking provide a new angle. CXCR2 antagonists have been verified in a range of preclinical cancer models as potential inhibitors to MDSCs recruitment, especially PMN-MDSCs (143, 144). Higher number of intratumoral CD33^+^ myeloid cells in patients with prostate cancer who relapse after docetaxel may suggest that MDSCs contribute to the resistance to chemotherapeutic drugs. In the same study, the resistance to docetaxel was reversed by treatment of an antagonist for CXCR2 performing as the inhibition of tumor growth and potentiation of chemotherapy-induced senescence (144). In murine rhabdomyosarcoma, high expression of surface PD-L1 were observed, and the anti-PD-1 therapy had limited efficacy in delayed treatment, compared to treatment early after tumor inoculation. Rhabdomyosarcoma induced the potent expansion of CXCR2^+^MDSCs in mouse model and a series of CXCR2 ligands in human pediatric sarcomas patients. The co-blockage of CXCR2 and PD-1 prevented MDSCs trafficking to the tumor, restored the anti-tumor effects of delayed ICIs treatment (143). In colorectal cancer, KRAS^*G*12*D*^-mediated suppression of IRF2 results in high expression of CXCL3, which binds to CXCR2 on MDSCs and promotes their recruitment into tumor site. The inhibition of CXCR2 overcome anti-PD-1 resistance to KRAS^*G*12*D*^-expressing tumors by suppressing KRAS^*G*12*D*^-driven MDSCs migration via the CXCL3/CXCR2 axis (145). For M-MDSCs, the enrichment of these cells are may insufficiently block by CXCR2 antagonists because of the expression of colony-stimulating factor 1 receptor (CSF1R) (144). CSF1 (CSF1R ligand) expression of melanoma and NSCLC cells is associated with the MDSC enrichment, which could be inhibited via the blockage of CSF1/CSF1R in vitro (146). Using a selective inhibitor BLZ945 to block the M-CSF/CSF1R interaction resulting in improved efficacy of PD-1 inhibitor in mice with neuroblastoma (147). Rebekka et al. showed that tumor-infiltrating M-MDSC were inhibited by CSF1R inhibitor PLX647 in B16 IDO-expressed melanoma mouse model, where the co-blockade of PD-1 and CTLA-4 were sensitized in via T cell activation (148). A research showed the same effect in colon and breast cancer mouse models, where the co-blockade of CTLA-4 and CSF1/CSF1R enhanced the beneficial effect by significant reduction in the number of tumor-infiltrating M-MDSCs, not PMN-MDSCs, and reprogramming M-MDSCs that displayed markedly increased expression of MHC class II and reduced expression of the immunosuppressive molecules ARG1 and TGF-β (146). The result from another study also supports this obversion that the blockage of CSF1/CSF1R signaling downregulated the population of M-MDSCs and TAM, reprogrammed the function of TAMs and DCs, and improved response to ICIs in pancreatic cancer model (149).

### ICIs Combining With an MDSC Functional Alteration

Entinostat (a histone-deacetylase inhibitor) eradicates 80% tumor and reduces MDSCs combining with CTLA-4 and PD-1 antibodies in different tumor-bearing mouse models, where the administration of each ICI alone is failed to induce anti-tumor response (150). Similar result has been shown in another study using mocetinostat, which the spectrum-selective inhibitor of class I/IV histone deacetylases in combination with PD-L1-targeting antibody has better benefit (151). Additionally, the reduction of the ARG1, iNOS and COX-2 levels in MDSCs induced by entinostat, synergize with the blockade of PD-1 significantly increasing survival in Lewis lung and RCC mouse models. Interestingly, the same study also reported that increased level of MDSCs in mice who received combination therapy treatment (152).

The mechanism of strategy that combining IDO inhibitors with ICIs remains unclear. Targeting Bruton’s tyrosine kinase (BTK) expressed in MDSCs in tumor-bearing mice by Ibrutinib, an irreversible inhibitor of BTK and IL2-inducible T-cell kinase which widely used for the treatment of B-cell malignancies in clinical, reduces the frequency of MDSCs in both the spleen and tumor. Ibrutinib also attenuated NO production and IDO expression of MDSCs, improved efficacy of anti-PD-L1 therapy (153). There is no direct evidence shows that IDO1 inhibitors enhance ICIs through the effects on MDSCs, but blocking IDO1 using its inhibitor INCB023843 in an anti-PD-1 resistant lung cancer mouse model has shown the reduction of IDO expression and MDSCs population, thereby delayed the tumor growth and metastasis (154). Combined therapy with IDO Inhibitors and ICIs seems to have improved depth and duration of responses in preclinical model (155, 156). Interestingly, a recent phrase ——— demonstrated that epacadostat (an IDO1 inhibitor) in combination with pembrolizumab showed no improvement of progression-free survival or overall survival in patients with advanced metastatic melanoma (157).

In various tumor-bearing mouse model, such as KRAS-mutant colorectal cancer (158, 159), BRAF^*V600E*^-mutant melanoma (160), and triple-negative breast cancer (161), the inhibition of MEK demonstrated better efficacy in combining with antibodies targeting PD-1 or PD-L1, compared to single agent. Recent study showed that the MEK inhibitors selumetinib could be a complement for anti-CTLA-4 therapy to negate the upregulation of COX-2 and ARG1 in the tumor after the neutralization of CTLA-4. The reduced percentage of CD11^+^Ly6G^+^myeloid cells and the accumulation of differentiating monocytes at the intermediate state with phenotype of Ly6C^+^MHC^+^ in tumor were induced by the combination therapy, thereby, enhancing the anti-tumor activity (162).

The vitamin A derivative all-trans retinoic acid (ATRA) induces the differentiation of immature myelocytic tumor cells in patients with acute promyelocytic leukemia resulting in death of the tumors cells. Similarly, ATRA acts on MDSCs to promote their differentiation and lead to decreased frequency of circulation MDSCs via ERK1/2 activation, glutathione synthase upregulation, and glutathione generation (163). In two previous clinical trials for patients with advanced RCC and lung cancer respectively, ATRA showed promising effect on reducing the frequency of MDSCs and promoting their differentiation into mature dendritic cells, macrophages, and granulocytes (23, 164). The combination of ATRA and cancer vaccine has improved patient response by depletion of MDSCs (164). The result from a very recent clinical trial that targets MDSCs using ATRA in melanoma patients treated with Ipilimumab is exciting. The significant reduction of the immunosuppressive genes NOX1, IL-10, TGFβ, IDO, and PD-L1 were induced by ATRA. Additionally, ATRA treatment also increased the expression of the MHC II transactivator which subsequently resulted in increased cell surface expression of HLA-DR, indicating a more differentiated state. ATRA plus Ipilimumab delayed the tumor progression in melanoma patients with similar frequency of serious adverse events compared to Ipilimumab-only treatment (165).

Considering about the VEGF playing an important role in MDSCs regulation and angiogenesis function, it can be a potential target to modulate the function of MDSCs. The blockade of VEGF, angiopoietin-2, and PD-1 significantly extended survival compared to vascular targeting alone in glioblastoma, which is non-T cell-inflamed cancer. The triple therapy increased the number of CTLs, which inversely correlated with MDSCs and Tregs, and showed significant global vascular normalization (166). Axitinib is a tyrosine kinase inhibitor targeting VEGFR1, 2, and 3. The combination therapy of axitinib with anti-CTLA-4 reduced tumor growth and increased survival in melanoma brain metastases models, both intracranial and subcutaneous. The same study showed the increased number of MDSCs with a higher ratio of M-MDSCs and PMN-MDSCs in both intracranial and subcutaneous model, where the reduction of suppression function of MDSCs and the enhanced antigen-presenting capacity of intratumoral DCs were also observed. Additionally, the combination therapy induced an antigen-presenting function of intratumoral M-MDSCs in subcutaneous tumor, not intracranial (167).

IPI-145, an inhibitor of phosphatidylinositol-4, 5-bisphosphate 3-kinase (PI3K)δ and PI3Kγ isoforms, suppressed PMN-MDSCs production of ARG1 and iNOS in a dose-dependent fashion. Although the high-dose IPI-145 treatment appeared to suppress TIL function, the combination of low dose IPI-145 and PD-L1 mAb may greater inhibit PMN-MDSCs than TIL to enhance responses to PD-L1 blockade (168). Using quinic acid (QA), another PI3Kδ/γ inhibitor, has been shown the same result in colon cancer model in combination with anti-PD-1 treatment (169).

### The Clinical Efficiency

Several results of clinical trials displayed uncertain efficiency of combining therapies. In advanced melanoma, a trial harboring 10 patients showed remarkable clinical benefits via the combination of ATRA and Ipilimumab. The average follow-time for the combination group is a year. During the follow-time, all of 4 patients in combination group have shown evidence of tumor progression, whereas 2 of 6 patients in Ipilimumab occur tumor progression proved by radiological evidence. All patients occurred headache, an expected side effect of ATRA, restoring after discontinuation of ATRA, however, the frequency of grade 3 or 4 adverse events are equal in two groups (165). Another study conducting in renal cell cancer indicated that the combining therapy (Axitinib plus pembrolizumab) can greatly improve the response rate in such patients. During 20.4 months median follow-time, patients had shown 73% objective response with median response time of 2.8 months in whole 52, containing 4 complete response and 34 partial response, and the median progression free survival time of 20 months. These result is much better in contrast to axitinib monotherapy (progression free survival time of 10–15 months) and nivolumab monotherapy (objective response of 13%). Tumor shrinkage was observed in 90% patients. Both PD-L1 positive expression patients and negative patients are beneficial from the combination therapy (170). Interestingly, in gastrointestinal stromal tumor and other sarcoma, the combination of dasatinib and ipilimumab seemed no enhance effect on each other. Nonetheless, study suggested the suppression of IDO expression may stabilize tumor progression. Using imatinib replace the dasatinib in these types cancer to combine anti-PD-1 is probably more effective, which drug also decreases both MDSCs and arginase 1 levels to normal ones (171, 172).

For hematological cancers, the trial of co-blockade is lack in clinical setting. One trial in stage 1b showed the MMR in chronic myeloid leukemia of 25% under the dose level1{*nivolumab 1 mg/kg q 2 weeks + dasatinib 100 mg QD (CP) or 140 mg QD (AP)*} and 45.5% under higher dose. However, most of the patients uncompleted the trail due to treatment failure (NCT02011945).

More ongoing clinical trial will offer more detail about the real value of these therapies in hematological cancer, such as NCT03516279, NCT02936752, NCT02332980 (Table 1).

## Further Direction

Myeloid-derived suppressor cells, as a highly heterogeneous cell type, is obviously restricted in further research of due to the lack of method to separate the neutrophils from PMN-MDSC. Also, the established standard of MDSCs is urgent. The future investigation may enlighten from biomarker representing unique function like the LOX-1, which is also associated of suppression function of MDSCs, the dominated character of MDSCs.

In this review, we highlight the powerful effect derived from complicated crosstalk within TME which is mediated by MDSCs. The regulations between MDSCs and Tregs are seemed one-way, showed in most of investigations. Nevertheless, several studies are challenging this notion. Adenosine, which we have mentioned as a T-cell suppression factor secreted by both MDSCs and Tregs, is a common inducer to MDSCs and Tregs and similar to COX−2, yes−associated protein 1 (YAP1) (173). Siret et al. illustrated the survival and proliferation of MDSCs is influenced by Tregs. The crosstalk between cells is realized via direct interactions and cell-to-cell way (174). In melanoma, Tregs modify the MDSCs to express higher level of B7-H1/3/4 and IL-10 (175). In these study, MDSCs and Tregs are compatible and mutually complementary in T cell suppression. As the studies of MDSCs are going, how the Tregs regulate the MDSCs induction, function need to pay more attention.

For clinical trial, many ongoing clinical trials aiming to different cancers through co-blockade of MDSCs and ICs, however, is not designed to realize its anti-tumor through such co-blockade. MDSCs have not been a conventional parameter for detect of immunoregulatory effect caused by combining therapies. The further trials should notice the effect on MDSCs while combing ICIs with treatment which interfered MDSCs, however, this interference may subordinate. Another one need to be emphasized is the role of combining therapies in hematological cancers, where the detail of triangular relationships among graft-versus leukemia effect, GVHD and MDSCs is unknown. In that domain, the insufficiency of studies started at pre-clinical model to clinical setting, where lack the more data for MDSCs after ICIs treatment. Besides several ongoing clinical trial, investigations of the role of MDSCs in that balance may more urgent and beneficial.

## Conclusion

Immune checkpoint inhibitors are the promising treatment approved for various cancer. The resistance occurring has limited their application for more patients. MDSCs accumulation derives a potent immunosuppressive network within tumor microenvironment and dysfunction T cell directly. Immune checkpoint inhibitors can be enhanced by combining with the therapies targeting MDSCs which break the net point of the network. The ongoing clinical trials (detail in Table 1) will provide more evidence about the safety and efficacy of these combination therapies.

## Author Contributions

AH contributed to the manuscript writing and revision, paper gathering and information analysis, and providing initial idea. KH contributed to the paper gathering and information analysis, and providing revision advice and checking. QH contributed to the paper gathering and information analysis, and providing revision advice. WC contributed to the manuscript writing and revision, paper gathering and information analysis, providing revision advice and checking, and replying to reviewers and editors. YL contributed to the revision, paper gathering and information analysis, and providing revision advice and checking.

## Conflict of Interest

The authors declare that the research was conducted in the absence of any commercial or financial relationships that could be construed as a potential conflict of interest.

## Abbreviations

cytotoxic T-lymphocyte-associated protein 4: CTLA-4
program death-1: PD-1
program death-1 ligand 1: PD-L1
dendritic cells: DC
major histocompatibility complex: MHC
human leukocyte antigen: HLA
macrophage colony-stimulating factor: M-CSF
peroxynitrite: PNT
nitric oxide: NO
a disintegrin and metallo proteinase domain 17: ADAM17
vascular endothelial growth factor: VEGF
signal transducer and activator of transcription: STAT
general control nonrepressed 2: GCN2
T cell immunoglobulin and mucin-domaincontaining molecules 3: TIM-3
cancer-associated fibroblasts: CAFs
semaphorins3A: Sema3A
neuropilin-1: Nrp1
lymphocyte activation gene 3: LAG-3
mitogen-activated protein kinase: MAPK
granulocyte-macrophage colony-stimulating factor: GM-CSF
granulocyte colony-stimulating factor: G-CSF
checkpoint kinase 1: CHK1
5-Fluorouracil: 5-FU
NOD-like receptor protein: Nlrp3
interferon regulatory factor 2: IRF2
Inducible nitric oxide synthase: iNOS
Cyclooxygenase-2: COX-2
mitogen-activated protein kinase kinase: MEK
extracellular regulated protein kinase: ERK
nicotinamide adenine dinucleotide phosphate oxidases 1: NOX1
small cell lung cancer: SCLC
rhabdomyosarcoma: RMS
colorectal cancer: CRC
pancreatic ductal adenocarcinoma: PDAC.
